# Effects of self-management programs on behavioral modification among individuals with chronic disease: A systematic review and meta-analysis of randomized trials

**DOI:** 10.1371/journal.pone.0254995

**Published:** 2021-07-23

**Authors:** Sunju Kim, Moonkyoung Park, Rhayun Song

**Affiliations:** 1 Sejong Chungnam National University Hospital, Daejeon, Korea; 2 College of Nursing, Chungnam National University, Daejeon, Korea; Edge Hill University, UNITED KINGDOM

## Abstract

The prevalence of chronic disease associated with unhealthy lifestyles has been increasing worldwide. Health professionals have recognized that self-management programs (SMPs) can provide health benefits by promoting health behaviors, especially when applied to individuals with lifestyle-related chronic disease. This review performed a meta-analysis of the features of SMPs using randomized studies and analyzed the magnitude of the combined effects of self-management on behavioral modification. We searched the PubMed, CINAHL, ScienceDirect, SCOPUS, Web of Science, Embase, Cochrane Library, DBpia, and KISS to identify randomized trials that evaluated the behavioral outcomes of SMPs. Subgroup analyses were performed for program duration, providers, type of comparisons, and program settings. We selected 25 studies (*N* = 5,681) to perform analyses with random-effects models. The effect sizes of SMPs were small but significant for physical activity (standardized difference in means [SDM] = 0.25), dietary habits (SDM = 0.28), and health responsibility (SDM = 0.18), and not significant for stress management and smoking behaviors. A short-term SMPs (less than 12 weeks) was indicated as being effective in modifying physical activity, dietary habits, and health responsibility, while the program effects on dietary habits were significant only with expert-delivered education and when compared with inactive controls. The findings of this study indicate that SMPs can effectively improve physical activity, dietary habits, and health responsibility in individuals with chronic disease, with a small but significant effect size. Future studies should explore the effects of SMPs on stress management and smoking cessation and assess the long-term maintenance of healthy lifestyles in individuals with lifestyle-related chronic disease.

## Introduction

Chronic disease associated with unhealthy lifestyle is currently the most common cause of mortality worldwide, accounting for approximately 31% of all deaths [1, 2]. Modifiable factors that contribute to the development of lifestyle-related chronic disease include smoking, high cholesterol, poor dietary habits, lack of physical activity, stress, and obesity [3]. Interventions for preventing lifestyle-related chronic diseases focus on managing modifiable cardiovascular risk factors, long-term behavioral modifications that increase physical activity, improving dietary habits, improving health responsibility and stress management, and cessation of smoking and alcohol consumption [4].

Among the various interventions for managing lifestyle-related chronic disease, a self-management program (SMP) based on an evidence-based disease prevention model focuses on behavioral modifications and self-management strategies in adults with chronic disease [5]. The goal of an SMP is to manage the health conditions of patients with chronic disease, improve their health status, and reduce their medical costs. Since most of the lifestyle-related chronic disease is dependent on self-management, behavioral modification through SMP is considered the most important treatment strategy. Most adults with lifestyle-related chronic disease, however, do not follow their recommended treatment regimens or maintain the modifications in their lifestyle in the long term [6–9]. Previous randomized controlled trials (RCTs) of patients with chronic disease and modifiable cardiovascular risk factors have consistently found that SMPs have positive effects on behavioral modifications, which leads to risk reduction and improved quality of life [10–12]. While a previous meta-analysis of SMPs mostly examined physiological outcomes for patients with chronic diseases [13], SMPs focus on the behavioral aspect of chronic disease management, for which those with lifestyle-related chronic disease have not been specifically assessed.

This study therefore aimed to identify randomized studies that used an SMP to improve the performance of health behaviors and to determine the size of the program’s combined effect on physical activity, dietary habits, health responsibility, stress management, and smoking in patients with lifestyle-related chronic disease and modifiable cardiovascular risk factors.

## Materials and method

### Search strategy

We searched for studies published from 2000 to December 2020 using international databases such as PubMed, CINAHL, ScienceDirect, SCOPUS, Web of Science, Embase, Cochrane Library, and Korean databases such as DBpia and KISS. This study conformed to the guidelines of the PRISMA (Preferred Reporting Items for Systematic Reviews and Meta-Analyses) [14]. The PICO (participants, interventions, comparisons and outcomes) framework was applied to our literature search. Search terms included various spellings and transliterations of self-management and lifestyle-disease keywords. The review protocol was registered at http://www.crd.york.ac.uk/PROSPERO (Registration number: CRD42021247523).

An example search strategy for PubMed was as follows: (((hypertension [MeSH Terms]) OR (diabetes [MeSH Terms]) OR (dyslipidemia [MeSH Terms]) OR (overweight [MeSH term]) OR (stroke [MeSH Terms]) OR (arthritis [MeSH Terms]) OR ((chronic* [All Fields]) AND (disease* [All Fields]))) NOT ((pulmonary disease, chronic obstructive [MeSH Terms]))) AND (((self [All Fields]) AND (management* [All Fields])) OR ((self management [All Fields]) AND support* [All Fields])) OR ((self [All Fields]) AND (regulation* [All Fields])) OR ((self [All Fields]) AND (monitoring [All Fields]))). Bibliographies of the retrieved articles national and international dissertations, conference proceedings and Google Scholar were used to perform manual searches for additional studies and grey literature.

### PICO framework

Adult participants (≥18 years old) with lifestyle-related chronic diseases and modifiable cardiovascular risk factors were included, such as diabetes, hypertension, hyperlipidemia, and obesity. The study intervention was an SMP alongside the basic mechanism of self-efficacy. The comparison was a control group provided with usual care, no treatment, health education, motivational interviewing, or group based exercise or mindful eating. The outcomes were specific health behaviors in association with modifiable cardiovascular risk factors, including physical activity, dietary habits, health responsibility, stress management, and smoking-related behavior [15].

The behavior modification activities that we focused on were everyday physical movements or regular exercise. Dietary habits referred to the extent to which the diet was healthy and balanced [16, 17]. Health responsibility referred to behavioral lifestyle changes with knowledge based personal and social responsibility, such as health care utilization, glucose monitoring, and medication adherence [18, 19]. Stress management involved managing stress to reduce cardiovascular disease and mortality risk [20]. Smoking-related behaviors comprised smoking cessation [21, 22] and tobacco dependence [23].

### Inclusion and exclusion criteria

The following inclusion criteria were developed: (1) RCTs on lifestyle-related chronic disease populations and published in English and Korean, (2) interventions conducted when an SMP was used regardless of its delivery mode or intensity, (3) presence of lifestyle-related chronic disease and modifiable cardiovascular risk factors such as diabetes, hypertension, hyperlipidemia, and obesity, and (4) assessments conducted on the behavioral outcomes of cardiac health behaviors (physical activity, dietary habits, health responsibility, stress management, and smoking). The exclusion criteria were (1) SMP conducted alongside other intervention programs, (2) inclusion of subjects with chronic diseases not related to modifiable cardiovascular risk factors, such as kidney disease, cancer, or arthritis, and (3) no pertinent data reported for the meta-analysis.

### Study selection (assessment of the risk of bias in the included studies)

Two reviewers (S.K. and R.S.) independently assessed the risk of bias in all included studies based on the Cochrane guidelines [24]. Reviewers were instructed to provide justifications for their assessments, to account for discrepancies, and to generate consensus ratings. Evaluated criteria included selection bias (random sequence generation and allocation concealment), performance bias (blinding of participants and personnel), detection bias (outcome assessment blinding), attrition bias (incomplete outcome data), reporting bias (selective reporting), and other sources of bias (baseline imbalance and differential attrition). Each criterion was rated as low, high, or unclear [24]. Any disagreements between the independent reviewers were resolved through group discussions.

### Data extraction

Two independent reviewers (S.K. and R.S.) extracted the data based on the Cochrane guidelines [24]. Extracted data included the mean and standard deviation (SD) of the pre- and posttest values in each group, the mean and SD values of changes in scores in each group, and the *t* score or *p* value within groups. Comprehensive Meta-Analysis Software (version 3) was used to estimate effect sizes (standardized differences in means [SDMs]) with 95% confidence intervals (CIs), and Egger’s regression test (*p*>.050) assessed publication bias with funnel plots as a reference. Heterogeneity was assessed using forest plots and *I*^2^ tests (>50%) to quantify inconsistencies in the included studies [25]. Effect sizes were calculated using a random-effects model as there were differences in disease type or composition and duration of the intervention [26].

According to the research purposes, a subgroup analysis was conducted to compare the program effects according to the program duration, program provider, the type of comparisons, and program settings.

## Results and discussion

### Participant characteristics and study settings

**Fig 1:**
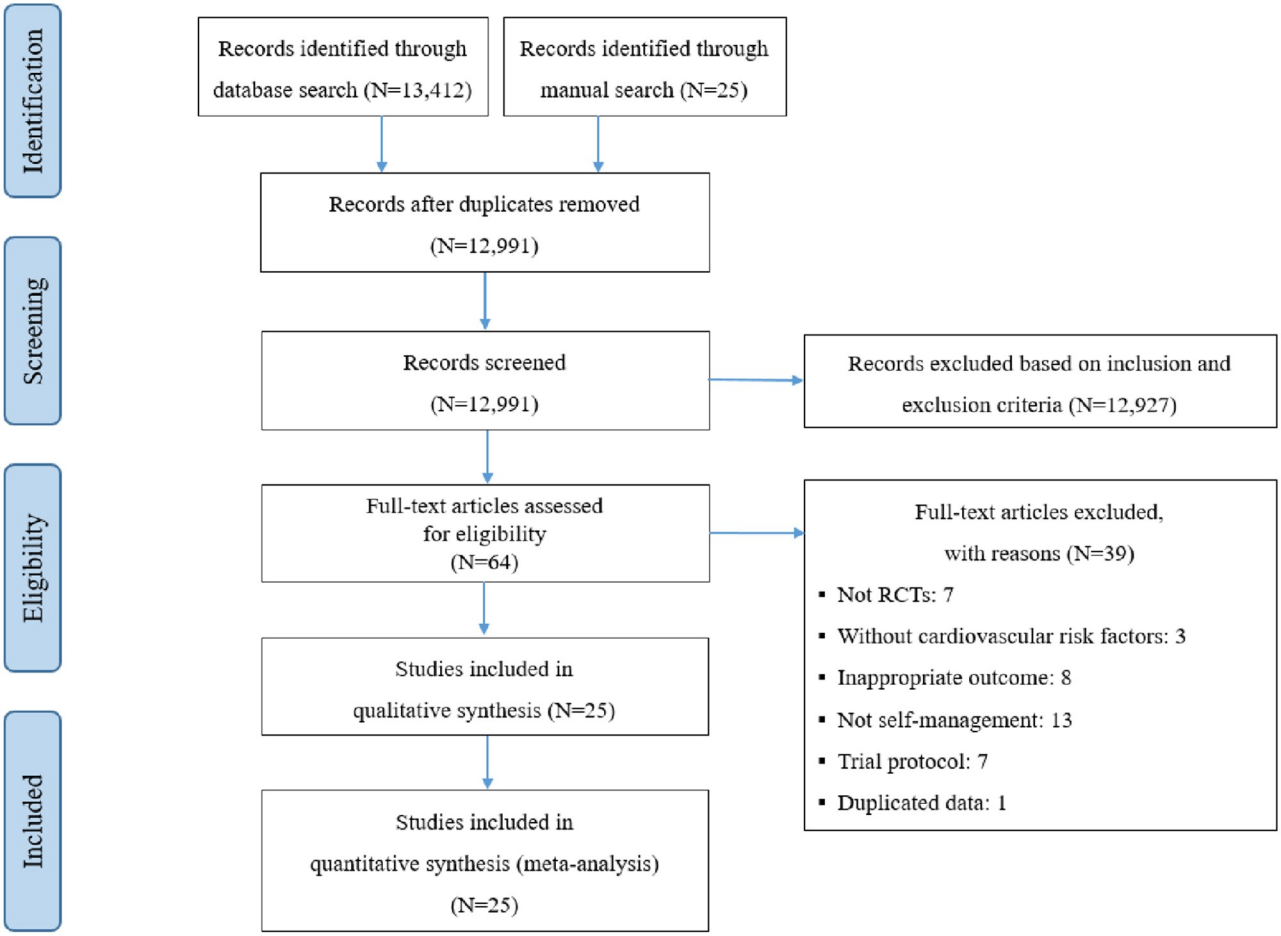
PRISMA flow diagram.

Our database searches identified 13,437 publications (5,095 studies on patients with lifestyle-related chronic disease) (Fig 1). Duplicate citations (*k* = 446) were removed using bibliographic software, leaving 12,991 citations for screening of the title and Abstract. After the initial screening of the inclusion and exclusion criteria, 64 full-text articles were reviewed. As listed in Table 1, 25 RCTs met the inclusion criteria and were included in the meta-analysis. All studies were published from 2003 to 2020. Nine studies were conducted in the USA, and the rest were conducted in China, Japan, Thailand, Australia, Canada, France, India, the Netherlands, Norway, and Canada. Participants were recruited from the community or a hospital. Among the cardiovascular risk factors, 14 studies applied SMPs to adults with diabetes, hypertension, hyperlipidemia, metabolic syndrome, or obesity.

**Table 1:** Qualitative description of included studies.

First author and Year	Country and setting	Health condition	*N* (E/C) Age, years (E/C)	Measurement time points	Intervention duration/ type/content	Intervention/ provider	Comparisons	Outcome variables			
Dongbo 2003	China Community	HTN, heart disease, DM	430/349 64.2/63.8	Baseline, 6M	7W/group 2–2.5h/W×7	CDSMP/peer	No treatment	PA (CDSMP)	HR (CDSMP)		
Steed 2005	UK Hospital	DM	65/59 59.2/60.3	Baseline, 3M, 6M	3M/group 2.5h×5+2.5h×1 (booster)	SMP/nurse, dietician	Usual care	Diet (SCDA)	Smoking cessation (SCDA)		
Hibbard 2007	USA Community	DM, HTN, heart disease	244/235 59.6/60.0	Baseline, 6W, 6M	Over 6W/group 2.5h×6	CDSMP/peer	No treatment	HR (PAM, health behaviors)	Stress management (health related behaviors)		
Lorig 2008	USA unity	DM	219/198 52.9/52.8	Baseline, 6M, 18M	6W/group 2.5h/W×6	SMP/peer	Usual care	PA (physical activities scale)	HR (glucose monitoring, health care utilization)		
Lorig 2009	USA Community	DM	186/159 67.7/65.4	Baseline, 6M, 12M	6W/group 2.5h/W×6	SMP/peer	Usual care	PA (health activity scale)	Diet (healthy eating)	HR (PAM, health care utilization)	
Moriyama 2009	Japan Hospital	DM	42/23 66.4/65.2	Baseline, 3M, 6M, 9M, 12M	12M/individual 30m×12 interview+1/2W call+interview	SMP/nurse	Usual care	PA (goal attainment for exercise)	Diet (goal attainment for diet)		
McGowan 2011	Canada Hospital	DM	82/152 55/59	Baseline, 6M	6W/group DM education 2.5h/W×6	CDSMP/peer	Regular DM education by a dietitian	PA (self-management behavior)	HR (medical care utilization)		
Rujiwatthanakorn2011	Thailand Hospital	HTN	50/46 61.2/60.9	Baseline, 14W	10W/group 2h×3+6 (letters)	SMP/primary investigator	Usual care	HR (SCABPCQ)	Stress management (SCABPCQ)		
Glasgow 2012	USA Hospital	DM	169 (E1)/162 (E2)/132 (C1) 58.7/58.7/58.7	Baseline, 4M, 12M	2M/(individual+group) 2h group+2 phone calls	Internet SMP /nutritionist	Usual care	PA (CHAMPS)	Diet (NCI percentage energy)	HR (Hill-Bone Compliance scale)	
Liu 2012	China Community	DM	119/89 62.0/62.5	Baseline, 12M	12M/(individual+group) 1.5h/M×12+1h/M×12 visits	CDSMP/doctor, nurse practitioner	Usual care	PA (self-management behavior)	Diet (self-management behavior)	HR (self-management behavior)	
Rygg 2012	Norway Hospital	DM	73/73 66	Baseline, 6M, 12M	3W/group **15h over 3** sessions	SMP/nurse	Usual care	HR (PAM)			
Suwankruhasn 2013	Thailand Hospital	Metabolic syndrome	44/42 59.6/62.7	Baseline, 3M, 6M	3M/group 2h/W×4+1/M×2	SMP/nurse	Usual care	PA (physical activity log)	Diet (7-day food diary)		
Trouilloud 2013	France Hospital	DM	120 56.7	Baseline, 3M	3 days/group 2–3h×8	SMP/nurse	No treatment	Diet (DM self-care activity)			
Forjuoh 2014	USA Community	DM	101/95 57.6	Baseline, 6M, 12M	6W/group 2.5h/W×6	CDSMP/peer	Usual care	Diet (DSCA)	HR (DSCA)		
Lynch 2014	USA Community	DM, HTN	31/30 54.8/53.4	Baseline, 6M	2W/group 3h×2+2h follow-up	SMP/community health worker, expert	Group education for nutrition and exercise	Diet (DSCA)	HR (MMTAS)		
Miller 2014	USA Community	DM	25/27 54.0/53.9	Baseline, 3M, 6M	3M/group 2.5h×10	SMP/dietitian	Group-based mindful eating intervention	Diet (BFFQ)			
Vinkers 2014	The Netherlands Community	Obesity	45/60 55.8/55.8	Baseline, 2M, 7M, 12M	6M/(individual+group) 1h×1, 2h×4+2h×2 (booster)	SMP/dieticians	Group session with written assignments	PA (PASE)	Diet (KFHQ)		
Whittle 2014	USA Community	HTN	219/185 68.8/67.4	Baseline, 12M	12M/group 10m×12	SMP/peer	Health education	PA (health survey)			
Baig 2015	USA Community	DM	50/50 51.7/55.7	Baseline, 3M, 6M	8W/group 90m/W×8	SMP/peer	Health education	PA (SDSCA)	Diet (SDSCA)	HR (SDSCA)	
Meng 2016	Germany Hospital	CHF	248/227	Baseline, 3M, 6M, 12M	3W/group 60–75m×5	SMP/physician, nurse, psychologist, physiotherapist	Education	PA (Godin Leisure-Time Exercise Questionnaire)	Diet (symptom monitoring: 4 items)	HR (MARS-D, symptom monitoring: 3 items)	
Sadeghian 2016	India Hospital	DM	152/154 45.4	Baseline 6M	2W/group 2h/W×2	SMP/expert	DM education	PA (questionnaire)	Smoking cessation (questionnaire)		
Nishimura 2017	Japan Hospital	DM	30/32 66.7/65.8	Baseline, 6M	6M/group 1/2M×3	SMP/health care staff	Usual care	HR (SDSCA)			
Huang 2018	China Hospital	HTN	46/44 54.6/54.5	Baseline, 1M, 3M, 6M	5W/(individual+group) 45m/W×3+motivational interviewing	SMP/nurse	Standard care plus routine health education	Diet (balance formulas)	HR (medication-taking behavior scale)		
Agarwal 2019	Canada Community	DM, HTN	28/22 64.2/63.9	Baseline, 4M	4M/individual 1/W×16 home visits+phone calls+HL app+discussion	SMP/peer	Usual care	PA (RAPA)			
Engelen 2020	The Netherlands Hospital	CVD	103/105 63.3/63.7	Baseline, 6M, 12M	12M/individual 6 modules×4	SMP/health professionals	Usual care	PA (IPAQ)	Diet (DHD Index)	HR (PAM)	Smoking (FTND)

Notes: *N*, population size; E, experimental group; C, comparison group; m, minutes; M, months; W, weeks; h, hours; PA, physical activity; HR, health responsibility; CDSMP, Chronic Disease Self-Management Program; SMP, self-management program; PAM, Patient Activation Measure; HTN, hypertension; PASE, Physical Activity Scale for the Elderly; KFHQ, Kristal Food Habits Questionnaire; BFFQ, Block 2005 Food Frequency Questionnaire; SCABPCQ, Self-Care Ability for Blood Pressure Control Questionnaire; SDSCA, Diabetes Self-Care Activities; SCDA, Self-Care Diabetes Activities; CHAMPS, Community Healthy Activities Model Program for Seniors; NCI, US National Cancer Institute; DSCA, Diabetes Self-Care Activities; MMTAS, Morisky Medication Adherence Scale; MARS-D, Medication Adherence Report Scale; HL app, healthy lifestyle application; RAPA, Rapid Assessment of Physical Activity; IPAQ, International Physical Activity Questionnaire; DHD Index, Dutch Healthy Diet Index; FTND, Fagerstrom Test for Nicotine Dependence; DM, diabetes mellitus; CHF, congestive heart failure; CVD, cardiovascular disease.

### Assessment of risk of bias in RCTs

**Fig 2:**
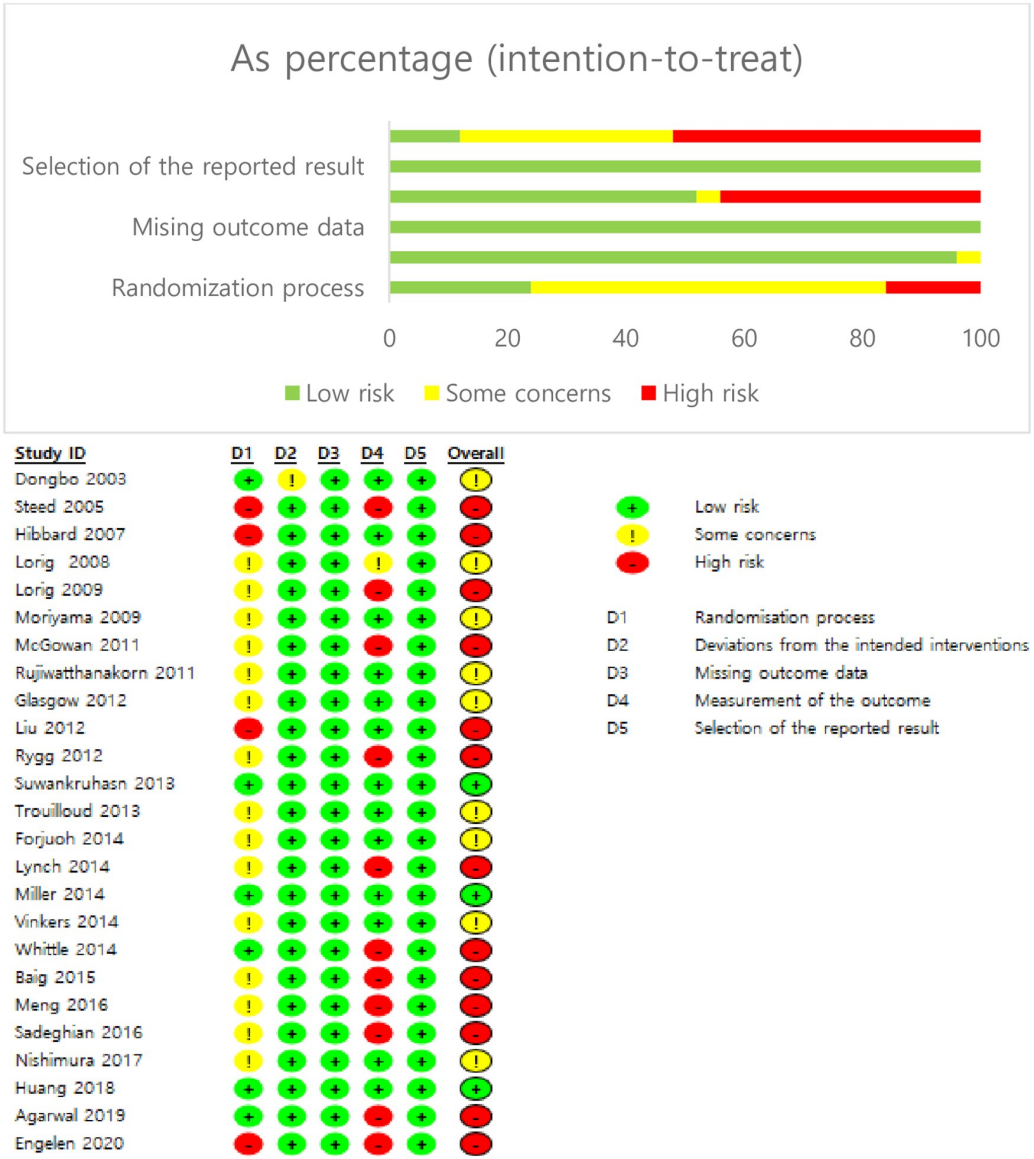
Summary of the risk of bias.

The risk of bias was low in all 25 RCTs (Fig 2). A specific random sequence or an allocation concealment process were reported for 18 (72%) and 16 (64%) studies, respectively. Participant and researcher blinding was impossible in most studies due to the characteristics of the RCTs, and blinding of the outcome assessments were reported for six studies (24%). All studies had a low dropout rate (<20%). The timing of outcome assessments was reported for all studies. All studies also had a low risk of selection bias. Our meta-analysis therefore included all 25 studies.

### Intervention and control group characteristics

Self-management interventions differed in their contents, durations, providers, and intensities (Table 1). The SMP sessions lasted from 10 to 180 minutes, and there were from 2 to 12 sessions. The overall SMP duration ranged from 3 days to 12 months. Each SMP was conducted on an individual basis (*k* = 18), group basis (*k* = 3), or a combination of both (*k* = 4). SMPs were delivered by either an expert (*k* = 16; health professionals, dieticians, or psychologists) or a peer (*k* = 9). The various components of SMP were considered, including education, interview, home visits, letters, phone calls, and discussions using health lifestyle apps.

The comparison groups mostly received the usual care or no treatment (*k* = 16), while nine studies considered group education as written assignments [27], mindful eating interventions [28], diabetes education [21, 29], health education [30, 31], or nutrition and exercise education [32].

### Primary analyses

**Fig 3:**
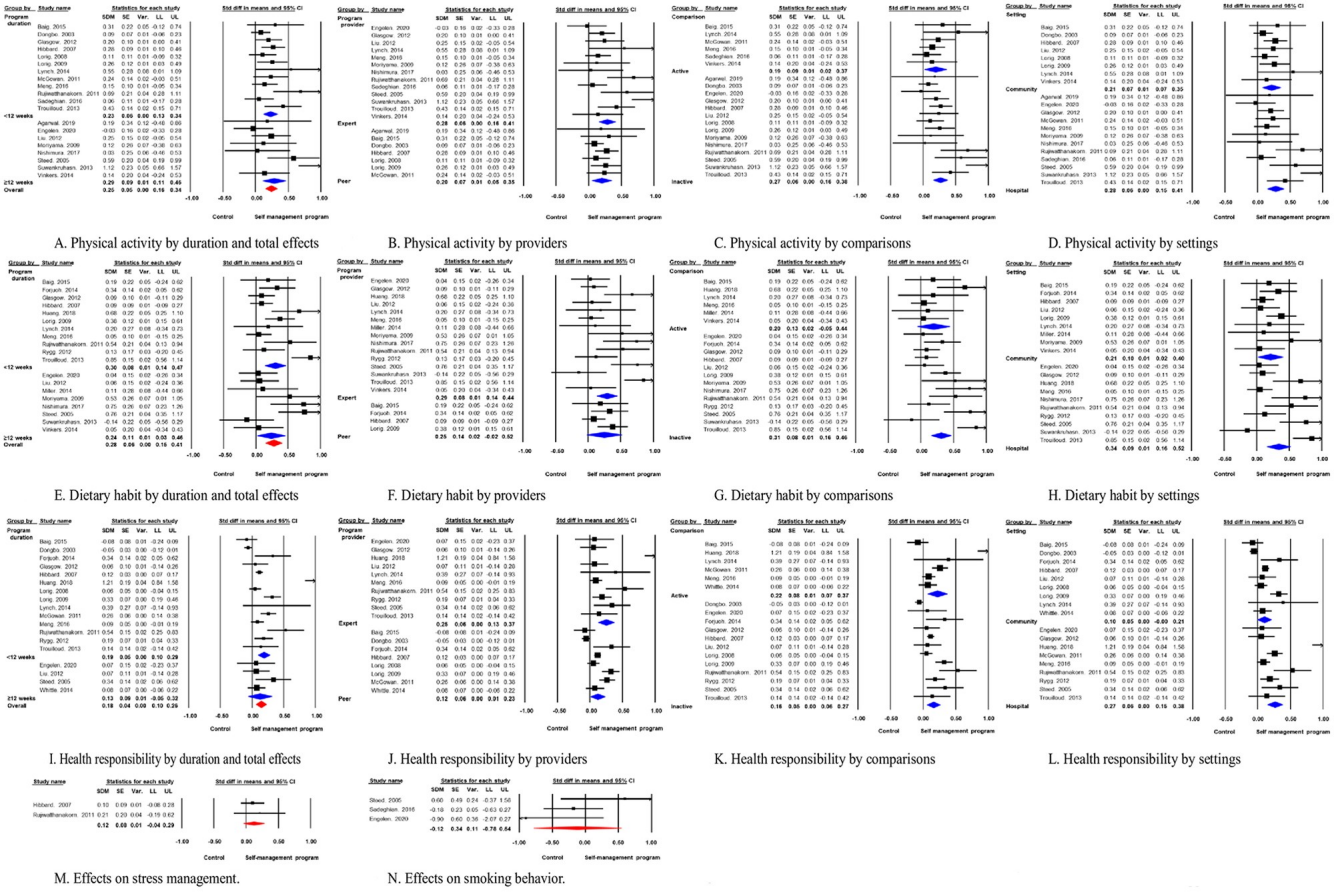
Forest plots of the effects of self-management programs (SMPs) on health behaviors. Notes: SDM, standardized difference in means; Cl, confidence interval; LL, lower limit; UL, upper limit.

Fig 3 shows the random-effects model, which calculated the effects of SMP on physical activity, dietary habits, health responsibility, stress management, and smoking. Subgroup analyses were conducted based on program duration (short term [<12 weeks] vs. long-term [≥12 weeks]), program provider (expert vs. peer), the type of comparisons (active control [education or physical activity] vs. passive control [usual care or no treatment]), and program settings (hospital vs. community).

#### Physical activities

Assessment of physical activity was an outcome of the SMP in 20 studies, as measured using physical activities scales [33, 34], exercise goal attainment [35], physical activity [36], the Physical Activity Scale for the Elderly [27], the Community Healthy Activities Model Program for Seniors [37], and a physical activity log book [38].

From the random-effects model, the pooled effect size of 20 studies for physical activities was small but significant (SMD = 0.25, 95% CI = 0.16~0.34, *I*^2^ = 49.5%) but potentially had publication bias (*p* = .039). The subgroup analyses were conducted for program duration, program provider, type of comparisons and program settings. The effect of short-term SMP (*k* = 12) was SMD = 0.23 (95% CI = 0.13~0.34, *I*^2^ = 29.9%) with potential publication bias (*p* = .008), while the effect of long-term SMP (*k* = 8) was SMD = 0.29 (95% CI = 0.11~0.46, *I*^2^ = 66.9%) with no publication bias (*p* = .587). No significant difference was found on the effect size of different intervention durations (Q = 0.25, *p* = .614) (Fig 3A).

Subgroup analyses on program providers revealed that the effect size of SMP on expert providers (*k* = 13) was SMD = 0.28 (95% CI = 0.16~0.41, *I*^2^ = 63.0%) with no publication bias (*p* = .094), while that of peer providers (*k* = 7) was SMD = 0.20 (95% CI = 0.05~0.35, *I*^2^ = 0.0%) with no publication bias (*p* = .354). The difference between the effect sizes of the program providers was not significant (Q = 0.75, *p* = .388) (Fig 3B).

Six of the 20 RCTs in which physical activity was assessed used active control for comparisons, while the other 14 compared SMP with usual care or no treatment controls. The effect size of the SMP on physical activity with active control was SMD = 0.19 (95% CI = 0.02~0.37, *I*^2^ = 0.0%) with potential publication bias (*p* = .079). When SMP was compared with usual care or no treatment control, the effect size was larger (SMD = 0.27, 95% CI = 0.16~0.38, *I*^2^ = 60.9%) with no publication bias (*p* = .105). The difference between the effect sizes of the control groups was not significant (Q = 0.55, *p* = .457) (Fig 3C).

Subgroup analysis on program settings revealed that the community based SMP (*k* = 8) had a smaller effect size (SMD = 0.21, 95% CI = 0.07~0.35, *I*^2^ = 0.0%) than the hospital based SMP (*k* = 7, SMD = 0.28, 95% CI = 0.15~0.41, *I*^2^ = 64.4%). No publication bias was suspected, and the difference between the program settings was not significant (Q = 0.45, *p* = .504) (Fig 3D).

#### Dietary habits

Fifteen studies assessed dietary habits by healthy eating [33], diet goal attainment [35], dietary activities [36], Kristal’s Food Habits Questionnaire [27], Block 2005 Food Frequency Questionnaire [28], questions on fatty food avoidance and vegetable intake [39], USA National Cancer Institute percent energy [37], and a 7-day food diary [38].

The total effect of SMPs on dietary habits was small but significant (SMD = 0.28, 95% CI = 0.15~0.41, *I*^2^ = 65.6%) in 19 studies with no publication bias (*p* = .117). Subgroup analyses by intervention duration showed that the effect of short-term SMP (*k* = 11) was SMD = 0.30 (95% CI = 0.14~0.47, *I*^2^ = 70.4%) with no publication bias (*p* = .121), while the effect of long-term SMP (*k* = 8) was SMD = 0.24 (95% CI = 0.03~0.46, *I*^2^ = 61.8%) with no publication bias (*p* = .218). The subgroup comparison revealed no significant differences (Q = 0.21, *p* = .647) (Fig 3E).

Most SMPs were provided by an expert (*k* = 15) with an effect size on dietary habits of SMD = 0.29 (95% CI = 0.14~0.44, *I*^2^ = 70.7%) with no publication bias (*p* = .152). Only four studies had a peer as the provider, and the effect size was not significant (95% CI = –0.02~0.52, *I*^2^ = 33.2%) with no publication bias (*p* = .594) (Fig 3F).

SMPs for dietary habits were mostly compared with inactive control (*k* = 13) with an effect size of SMD = 0.31 (95% CI = 0.16~0.47, *I*^2^ = 72.2%) and no publication bias (*p* = .141). The effect size of the SMP with active control (*k* = 6) was not significant (95% CI = –0.05~0.44, *I*^2^ = 31.1%) with no publication bias (*p* = .347) (Fig 3G).

Subgroup analysis on program settings revealed that the community based SMP (*k* = 9) had a smaller effect size (SMD = 0.21, 95% CI = 0.02~0.40, *I*^2^ = 0.0%) than the hospital based SMP (*k* = 10, SMD = 0.34, 95% CI = 0.16~0.52, *I*^2^ = 79.4%). No publication bias was suspected, and the difference between the program settings was not significant (Q = 0.93, *p* = .334) (Fig 3H).

#### Health responsibilities

Health responsibilities were an outcome in 16 studies, which included glucose monitoring [33, 34], health care utilization [29, 33, 34, 39, 40], communication with physician [33, 34], food label reading [33], feet inspection [39], medication adherence [39], Patient Activation Measure [10, 33, 39], medication adherence [36], Hill-Bone Compliance to High Blood Pressure Therapy Scale [37], and Morisky Medication Adherence Scale [32].

The total effect size of SMPs on health responsibility (*k* = 18) was small but significant (SMD = 0.18, 95% CI = 0.10~0.26, *I*^2^ = 82.8%) with potential publication bias (*p* = .032). The subgroup analysis indicated that the effect size of the short-term SMP on health responsibility (*k* = 14) was SMD = 0.19 (95% CI = 0.10~0.29, *I*^2^ = 86.4%) but there was potential for publication bias (*p* = .040). Only four studies compared the long-term SMP, which had an insignificant effect size (95% CI = –0.05~0.32, *I*^2^ = 0.8%) and no publication bias (*p* = .481) (Fig 3I).

SMPs delivered by expert providers (*k* = 10) had a small but significant effect size of SMD = 0.25 (95% CI = 0.13~0.37, *I*^2^ = 79.5%) with no publication bias (*p* = .082). The SMP effect size with peer providers (*k* = 8) was small but significant (SMD = 0.12, 95% CI = 0.01~0.23, *I*^2^ = 85.5%) with no publication bias (*p* = .429). There were no significant differences in effect sizes between the program provider groups (Q = 2.49, *p* = .115) (Fig 3J).

The SMPs utilizing active control (*k* = 6) tended to have larger effect sizes (SMD = 0.22, 95% CI = 0.07~0.37, *I*^2^ = 89.1%) than those with passive control (*k* = 12) (SMD = 0.16, 95% CI = 0.06~0.27, *I*^2^ = 76.8%), and had no publication bias (*p* = .262, *p* = .107, respectively). No significant difference on effect size was found between these groups (Q = 0.42, *p* = .519) (Fig 3K).

The subgroup analysis on program settings indicated that the community based SMP (*k* = 9) was not significant (95% CI = 0.00~0.21, *I*^2^ = 80.2%), while the hospital based SMP (*k* = 7) has a significant small effect size (SMD = 0.27, 95% CI = 0.15~0.38, *I*^2^ = 81.7%). No publication bias was suspected (*p* = .124) (Fig 3L).

#### Stress management

Only two studies assessed the effects of SMPs on stress management, which was measured using 18 health-related behaviors [10] and SCABPCQ [41]. The effect size for SMPs on stress management was not significant (SDM = 0.12, 95% CI = –0.04~0.29) and had low heterogeneity (*I*^2^ = 0.0%) (Fig 3M).

#### Smoking behavior

Only three studies assessed the effects of SMPs on smoking behavior, which was measured by a smoking cessation questionnaire [21, 22] or the Fagerstrom Test for Nicotine Dependence [23]. The random-effects model indicated that the SMP effect size on smoking behavior was not significant (SDM = –0.12, 95% CI = –0.78~0.54) and had moderate heterogeneity (*I*^2^ = 48.5%) and no publication bias (*p* = .999) (Fig 3N).

## Discussion

This systematic review and meta-analysis evaluated 25 RCTs that used SMPs and were performed worldwide, and found that SMPs were effective on behavioral modification, specifically physical activity, dietary habits, and health responsibility. There were no significant effects on stress management and smoking behavior due to the relatively small number of studies that assessed these behavioral outcomes. Several parameters varied in the SMPs promoting behavioral modification for individuals with lifestyle-related chronic disease, including control conditions, intervention durations, program providers, and program settings. We used a random-effects model to consider the assumption that different studies estimated different treatment effects from SMP [26].

An SMP has been supported as an effective intervention to improve medication adherence, which we defined as health responsibility [42]. Our findings are similar to those in previous meta-analyses, which found SMPs to have significant effects on health responsibilities, including improved medication adherence and decreased use of hospitals for asthma and stroke patients [43]. However, no significance differences in stress management and smoking behaviors were found between the experimental and control groups, which is likely due to the relatively small number of studies that we analyzed.

The SMP intervention duration ranged from 3 days to 12 months with a frequency range of 3 to 12 sessions. Most of the included studies (*k* = 14) applied a short-term SMP with a cutoff of less than 12 weeks, and had a small but significant effect size for improving physical activity, dietary habits, and health responsibility. Our meta-analysis indicated that the effect size of long-term SMPs was similar to of short-term SMPs except for health responsibility, suggesting that SMP lasting for up to 12 weeks can effectively modify the behaviors of individuals with cardiovascular risk factors. Previous meta-analyses of chronic disease SMPs with durations of 4~6 months found small but significant effect sizes on aerobic exercise (SMD = 0.12) and health responsibility (communication with physician: SMD = 0.26) [44]. In the current analysis, the long-term effect of SMPs on health responsibility was not significant, probably due to the small number of the studies (k = 4) included in the meta-analysis.

In our subgroup analysis on the type of comparisons, SMPs was effective to promote physical activity and health responsibilities, regardless of using active or inactive controls. However, the SMPs applied to modify dietary habits was only effective when compared with inactive controls. Only 6 studies applied active controls to compare the effect of SMPs on dietary habits, which could be the reason for not significant effects.

The effects of expert- and peer-delivered programs on physical activity, dietary habits, and health responsibility were also compared. Previous research indicated that health education provided by experts from multidisciplinary teams had large effects on behavioral modification [45]. However, the effects of the SMPs when they were delivered by experts was not significantly different when compared with peer-delivered programs for physical activity and health responsibility. The SMPs was effective on dietary habits only when delivered by experts (e.g., dietitians or health professionals).

The effects of the SMP may vary according to the program settings. In our meta-analysis, both hospital-based and community-based SMPs were found significant on improving physical activities and dietary habits, but only the effect of hospital-based SMPs was significant on health responsibility. Health responsibility as behavioral outcome refers to health care utilization, glucose monitoring, or medication adherence. Previous meta-analysis on lay-led self-management program [46] also found the mixed results regarding appropriate health care utilization, in that the SMP approach may encourage the participants to seek medical advice or could reduce the unplanned hospital visits.

There were several limitations in this systematic review and meta-analysis. First, we examined the effects immediately after interventions and did not include follow-up data. There are currently insufficient follow-up data for examining the long-term effects of SMPs on behavior modification. Further studies should consider follow-up data and analyze the long-term retention of SMP effects on behavioral modification, which may improve the management of lifestyle-related chronic disease. Second, only studies in English and Korean were included, which may reduce the generalizability of our findings. Third, relatively few randomized studies were available that analyzed stress management and smoking-related behaviors, and so further studies are necessary to evaluate the effect of SMP on these specific health behaviors based on delivery mode (group vs. individual, online vs. offline, and program intensity).

## Conclusions

The findings of this study indicate that SMPs can effectively improve physical activity, dietary habits, and health responsibility in individuals with lifestyle-related chronic disease, with a small but significant effect size. A short-term SMPs (less than 12 weeks) was indicated as being effective in modifying physical activity, dietary habits, and health responsibility, while the program effects on dietary habits were significant only with expert-delivered education and when compared with inactive controls. Further studies are warranted with larger trials and more rigorous methodologies that explore the effects of SMPs on stress management and smoking cessation and examine long-term outcomes of the SMPs.

## Supporting information

S1 Checklist (DOCX)

S1 File (PDF)
