# Supplementary material for: Effects of self-management programs on behavioral modification among individuals with chronic disease: A systematic review and meta-analysis of randomized trials
Source: PLoS One. 2021 Jul 23;16(7):e0254995. doi: 10.1371/journal.pone.0254995 (PMC8301623; doi:10.1371/journal.pone.0254995)
Supplement: S1 File — (PDF) [file pone.0254995.s002.pdf]

Study name	provider	comparison	outcomes	time point	setting	data format	mean change(ex)	p for change(ex)	sample size(ex)	mean change(cont.)	p for change(cont.)	sample size(cont)				
Miller. 2014	expert	active	Dairy(Servings/1,000 kcal)	short	Community	Change, p in each group	0.06	0.842	25	0.02	0.82	27				
	expert	active	Fats/Oils, Sweets, Soda(Servings/1,000 kcal)	short	Community	Change, p in each group	0.05	0.701	25	-0.04	0.757	27				
	expert	active	Fruits and fruit juices(servings/1000kcal)	short	Community	Change, p in each group	0.38	0.0095	25	0.27	0.0493	27				
	expert	active	Grains(servings/1000kcal)	short	Community	Change, p in each group	-0.37	0.0703	25	-0.03	0.8903	27				
	expert	active	Meat,Fish,Poultry,Eggs(servings/1000kcal)	short	Community	Change, p in each group	0.1	0.336	25	0.06	0.7203	27				
	expert	active	vegetables(servings/1000kcal)	short	Community	Change, p in each group	0.8	0.0035	25	0.24	0.0224	27				
							sample size(ex)	sample size(cont)	F or diff.							
Hibbard. 2007	peer	inactive	pay attention to amount of fat in diet	short	Community	F for diff in change	244	235	0.99							
Moriyama. 2009	expert	inactive	Dietary stage	long	Community	F for diff in change	42	23	4.171							
							diff. in means	sample size(ex)	sample size(cont)	independent groups p						
Lynch. 2014	expert	active	carbohydrate	short	Community	Independent groups (difference, p)	1.7	29	26	0.64						
	expert	active	cholesterol	short	Community	Independent groups (difference, p)	-53	29	26	0.45						
	expert	active	dietary intake	short	Community	Independent groups (difference, p)	-247.9	29	26	0.74						
	expert	active	dietary sodium	short	Community	Independent groups (difference, p)	-475.9	29	26	0.64						
	expert	active	fat intake	short	Community	Independent groups (difference, p)	-0.4	29	26	0.34						
	expert	active	fiber	short	Community	Independent groups (difference, p)	-2.3	29	26	0.16						
	expert	active	general diet	short	Community	Independent groups (difference, p)	0.5	29	26	0.001						
	expert	active	potassium	short	Community	Independent groups (difference, p)	-375.6	29	26	0.32						
	expert	active	protein intake, % of total intake	short	Community	Independent groups (difference, p)	-1.1	29	26	0.01						
	expert	active	special diet	short	Community	Independent groups (difference, p)	0.3	29	26	0.02						
	expert	active	vegetable	short	Community	Independent groups (difference, p)	-0.4	29	26	0.05						
							mean(ex)	sample size(ex)	mean(cont.)	sample size(cont)	p					
Rujiwatthanakorn. 2011	expert	inactive	Dietary control	short	Hospital	Independent groups (means, p)	55.04	50	41.39	46	0.01					
Forjuoh. 2014	peer	inactive	Eat ≥ 5 fruit/vegetable servings	short	Community	Independent groups (means, p)	3.45	101	3.95	95	0.02					
							sample size(ex)	sample size(cont)	independent groups p							
Rygg. 2012	expert	inactive	avoidance of fatty foods	short	Hospital	Independent groups (Sample size, p)	73	73	0.885							
	expert	inactive	high vegetable intake	short	Hospital	Independent groups (Sample size, p)	73	73	0.168							
							mean difference(ex)	difference SD(ex)	sample size(ex)	mean difference(cont.)	difference SD(cont.)	sample size(cont)				
Lorig. 2009	peer	inactive	Healthy eating (0-6) ↑	short	Community	Mean change, SD difference in each group	0.198	0.75	161	-0.082	0.718	133				
Liu. 2012	expert	inactive	Eating fatty foods (grams per day)	long	Community	Mean change, SD difference in each group	-0.06	15.76	98	0.92	6.6	78				
	expert	inactive	Eating fruits (pieces per day)	long	Community	Mean change, SD difference in each group	0.12	0.61	98	0.16	0.91	78				
	expert	inactive	Eating vegetables (grams per day)	long	Community	Mean change, SD difference in each group	22.94	154.43	98	-10.26	165.75	78				
Vinkers. 2014	expert	active	diet	long	Community	Mean change, SD difference in each group	-0.03	1.66	45	-0.12	2.13	60				
Nishimura. 2017	expert	inactive	change in diet subscale	long	Hospital	Mean change, SD difference in each group	-0.03	1.16	30	0.83	1.14	32				
Huang. 2018	expert	active	sodium intake	short	Hospital	Mean change, SD difference in each group	1.7	3.7	46	-0.1	0.34	44				
							pre mean(ex)	pre SD(ex)	post mean(ex)	post SD(ex)	sample size(ex)	pre mean(cont.)	pre SD(cont.)	post mean(cont.)	post SD(cont.)	sample size(cont.)
Suwankruhasn. 2013	expert	inactive	carbohydrate	short	Hospital	Means, SD in each group	58.01	11.47	61.83	10.48	44	60.43	11.71	59.62	9.76	42
	expert	inactive	cholesterol	short	Hospital	Means, SD in each group	216.2	158.3	203.7	176.47	44	203.69	168.76	209.8	163.82	42
	expert	inactive	fat intake	short	Hospital	Means, SD in each group	26.03	9.84	22.74	8.31	44	23.69	9.73	24.21	8.08	42
	expert	inactive	Fiber	short	Hospital	Means, SD in each group	9.27	4.78	9.28	7.57	44	9.05	5.29	9.98	6.54	42
	expert	inactive	fiber	short	Hospital	Means, SD in each group	9.27	4.78	9.28	7.57	44	9.05	5.29	9.98	6.54	42
	expert	inactive	protein	short	Hospital	Means, SD in each group	15.99	4.21	15.44	4.49	44	16.43	5.28	16.14	4.63	42
	expert	inactive	sodium	short	Hospital	Means, SD in each group	2000	1375.35	1853	908.77	44	2190.83	1262.3	2252	1213.28	42
	expert	inactive	sugar	short	Hospital	Means, SD in each group	38.56	31.24	32.03	21.44	44	37.71	30.74	43.21	28.57	42
Trouilloud. 2013	expert	inactive	Dietary behaviors	short	Hospital	Means, SD in each group	3.22	2.74	5.54	2.51	99	3.33	2.77	3.86	1.59	99
Steed. 2005	expert	inactive	Diet.general	short	Hospital	Means, SD in each group	4.94	1.78	5.67	1.18	50	5.07	2.16	4.94	1.93	50
	expert	inactive	Diet-specific diet	short	Hospital	Means, SD in each group	4.35	1.72	5.34	1.48	50	4.84	1.71	4.32	1.58	50
Glasgow. 2012	expert	inactive	Eating Havits(score;range=1/worst-3/best)	short	Hospital	Means, SD in each group	2.18	0.364	2.31	0.182	331	2.13	0.345	2.18	0.23	132
	expert	inactive	Fat Intake1 (%; range=20.50)	short	Hospital	Means, SD in each group	34.86	5.094	33.71	4.912	331	35.18	4.596	35.11	4.711	132
Baig. 2015	peer	active	Eating 5 or more servings of fruits and vegetables	short	Community	Means, SD in each group	3.15	2.52	4.49	2.38	43	3.95	2.48	5.23	2.19	41
	peer	active	Following a healthful eating plan	short	Community	Means, SD in each group	2.65	2.73	3.8	2.78	43	4.56	2.51	4.73	2.74	41
Engelen. 2020	expert	inactive	Dutch health diet index	long	Hospital	Means, SD in each group	56	12	55.5	9.3	78	57.1	11.1	57	10.8	96
Meng. 2016	expert	active	prevention diet	short	Hospital	Means, SD in each group	12.07	2.46	12.86	2.32	207	12.43	2.52	13.1	2.39	193

Study name	provider	comparison	outcomes	time point	setting	data format	sample size(ex)	sample size(cont)	F or diff.							
Hibbard. 2007	peer	inactive	Able to maintain recommended weight	short	Community	F for diff in change	244	235	1.4							
Hibbard. 2007	peer	inactive	Ask physician or pharmacist about medication side effects	short	Community	F for diff in change	244	235	0.99							

Hibbard. 2007	peer	inactive	Check BP at least once a week	short	Community	F for diff in change	244	235	3.6							
Hibbard. 2007	peer	inactive	Check feet for cracks and calluses	short	Community	F for diff in change	244	235	1.3							
Hibbard. 2007	peer	inactive	Keep written diary of BP readings	short	Community	F for diff in change	244	235	0.99							
Hibbard. 2007	peer	inactive	Keep written diary of glucose levels	short	Community	F for diff in change	244	235	1.1							
Hibbard. 2007	peer	inactive	Read about side effects when prescribed new medication	short	Community	F for diff in change	244	235	0.99							
Hibbard. 2007	peer	inactive	Read food labels for content	short	Community	F for diff in change	244	235	0.99							
Hibbard. 2007	peer	inactive	Take BP medications as physician recommends	short	Community	F for diff in change	244	235	0.99							
Hibbard. 2007	peer	inactive	Taking diabetes medication	short	Community	F for diff in change	244	235	9.2							
Hibbard. 2007	peer	inactive	Test glucose at least three times a week	short	Community	F for diff in change	244	235	0.99							
							diff. in means	sample size(ex)	sample size(cont)	independent groups p						
Lynch. 2014	expert	active	glucose testing	short	Community	Independent groups (difference, p)	-0.3	29	26	0.15						
							mean(ex)	sample size(ex)	mean(cont.)	sample size(cont)	p					
Rujiwatthanakorn. 2011	expert	inactive	medication taking	short	Hospital	Independent groups (means, p)	54.88	50	41.57	46	0.009					
Rujiwatthanakorn. 2011	expert	inactive	self monitoring	short	Hospital	Independent groups (means, p)	54.25	50	42.25	46	0.011					
Forjuoh. 2014	peer	inactive	Check your feet	short	Community	Independent groups (means, p)	4.61	101	5.48	95	0.02					
							sample size(ex)	sample size(cont)	independent groups p							
Lorig. 2009	peer	inactive	Glucose monitoring	short	Community	Independent groups (Sample size, p)	161	133	0.001							
Rygg. 2012	expert	inactive	Oral glucose lowering agent and/or insulin	short	Hospital	Independent groups (Sample size, p)	73	73	0.891							
Rygg. 2012	expert	inactive	self-inspection of feet	short	Hospital	Independent groups (Sample size, p)	73	73	0.147							
Rygg. 2012	expert	inactive	self-monitor of blood glucose	short	Hospital	Independent groups (Sample size, p)	73	73	0.131							
Rygg. 2012	expert	inactive	Visit, health care personnel past 3 months	short	Hospital	Independent groups (Sample size, p)	73	73	0.106							
							mean difference(ex)	difference SD(ex)	sample size(ex)	mean difference(cont.)	difference SD(cont.)	sample size(cont)				
Lorig. 2009	peer	inactive	Communication with physician (0-5) ↑	short	Community	Mean change, SD difference in each group	0.31	0.992	161	0.068	0.934	133				
Lorig. 2009	peer	inactive	days in hospital(past 6M)	short	Community	Mean change, SD difference in each group	0	4.31	161	-0.143	1.75	133				
Lorig. 2009	peer	inactive	Emergency visit(past 6M)	short	Community	Mean change, SD difference in each group	-0.012	0.742	161	0.113	0.885	133				
Lorig. 2009	peer	inactive	Patient activation (PAM) (0-100) ↑	short	Community	Mean change, SD difference in each group	4.52	15.8	161	1.75	15.3	133				
Lorig. 2009	peer	inactive	physiciam visit(past 6M)	short	Community	Mean change, SD difference in each group	0.219	3.88	161	-0.106	3.37	133				
Lorig. 2009	peer	inactive	Read food labels (0-4) ↑	short	Community	Mean change, SD difference in each group	0.119	0.941	161	-0.18	0.824	133				
Dongbo. 2003	peer	inactive	Communication with medical doctor	short	Community	Mean change, SD difference in each group	0.04	1.24	396	0.11	1.32	308				
Dongbo. 2003	peer	inactive	Emergency room visits	short	Community	Mean change, SD difference in each group	-0.04	0.96	430	-0.03	0.72	349				
Dongbo. 2003	peer	inactive	Hospital stays	short	Community	Mean change, SD difference in each group	-0.06	0.46	430	0.06	0.92	348				
Dongbo. 2003	peer	inactive	Nights in hospital	short	Community	Mean change, SD difference in each group	-0.55	9.6	430	0.44	6.72	349				
Dongbo. 2003	peer	inactive	Physician visits	short	Community	Mean change, SD difference in each group	-1.01	9.41	430	-0.84	7.76	349				
Liu. 2012	expert	inactive	Communication with medical doctor	long	Community	Mean change, SD difference in each group	0.41	1.54	98	0.22	1.09	78				
Liu. 2012	expert	inactive	Examining feet (times per week)	long	Community	Mean change, SD difference in each group	0.46	2.08	98	0.45	2.86	78				
Lorig. 2008	peer	inactive	communication with physician(0-5)↑	short	Community	Mean change, SD difference in each group	0.324	1.45	179	0.144	1.34	173				
Lorig. 2008	peer	inactive	days in hospital	short	Community	Mean change, SD difference in each group	0.354	7.18	179	-0.087	1.49	173				
Lorig. 2008	peer	inactive	emergency visits	short	Community	Mean change, SD difference in each group	-0.107	0.82	179	-0.081	0.943	173				
Lorig. 2008	peer	inactive	physician visits	short	Community	Mean change, SD difference in each group	-0.028	3.14	179	-0.064	2.64	173				
Lorig. 2008	peer	inactive	test glucose	short	Community	Mean change, SD difference in each group	0.05	0.387	179	0.08	0.365	173				
Whittle. 2014	peer	active	PAM <53 (levels 1 or 2)	long	Community	Mean change, SD difference in each group	-2.6	22.198	219	-5.8	23.122	185				
Whittle. 2014	peer	active	PAM ≥53 (levels 3 or 4)	long	Community	Mean change, SD difference in each group	-4.6	25.158	219	-5	24.483	185				
Huang. 2018	expert	active	medication adherence	short	Hospital	Mean change, SD difference in each group	39	134.95	46	7.2	89.68	44				
Huang. 2018	expert	active	RHBP	short	Hospital	Mean change, SD difference in each group	37.5	7.445484223	46	2.2	7.44	44				
							pre mean(ex)	post mean(ex)	sample size(ex)	pre mean(cont.)	post mean(cont.)	sample size(cont.)	F for diff.			
Hibbard. 2007	peer	inactive	patient activation measure	short	Community	Means, F for diff in change	59.9	64.6	244	59.8	63.1	235	2.344			
							pre mean(ex)	pre SD(ex)	post mean(ex)	post SD(ex)	sample size(ex)	pre mean(cont.)	pre SD(cont.)	post mean(cont.)	post SD(cont.)	sample size(cont.)
Trouilloud. 2013	expert	inactive	adherence to medication	short	Hospital	Means, SD in each group	6.4	0.97	6.39	0.8	99	6.53	1.27	6.69	1.47	99
Steed. 2005	expert	inactive	Foot care	short	Hospital	Means, SD in each group	3.33	2.32	3.98	2.24	50	3.39	2.32	3.79	2.41	50
Steed. 2005	expert	inactive	HBGM(blood glucose monitoring)	short	Hospital	Means, SD in each group	3.38	3.1	5.07	1.97	50	2.67	2.72	2.94	2.8	50
Glasgow. 2012	expert	inactive	medication adherence dichotomized	short	Hospital	Means, SD in each group	0.35	0.546	0.42	0.546	331	0.34	0.46	0.38	0.46	132
McGowan. 2011	peer	active	Communication with physician (0-5) ↑	short	Hospital	Means, SD in each group	2.62	1.12	2.96	1.07	82	2.4	1.02	2.26	1.12	152
McGowan. 2011	peer	active	Number of doctor visit	short	Hospital	Means, SD in each group	3.2	2.09	2.35	2.3	82	3.34	2.37	2.62	2.31	152
McGowan. 2011	peer	active	Number of times hospitalized for 1 night or longer	short	Hospital	Means, SD in each group	0.05	0.28	0.01	0.12	82	0.04	0.21	0.1	0.34	152
McGowan. 2011	peer	active	Number of visits to emergency room	short	Hospital	Means, SD in each group	0.15	0.49	0.07	0.38	82	0.17	0.48	0.18	0.61	152
McGowan. 2011	peer	active	Total number of nighrs spent in hospital	short	Hospital	Means, SD in each group	0.4	1.99	0.17	1.39	82	0.1	0.5	0.48	2.17	152
Baig. 2015	peer	active	Testing your blood sugar the number	short	Community	Means, SD in each group	2.08	2.86	3.95	2.85	43	2.07	2.66	4.05	2.89	41
Baig. 2015	peer	active	Checking your feet	short	Community	Means, SD in each group	4.56	3.07	5.73	2.21	43	5.54	2.72	6.44	1.69	41
Baig. 2015	peer	active	Days per week on average following eating plan over last month	short	Community	Means, SD in each group	2.93	2.93	3.88	2.87	43	4.27	2.47	4.76	2.57	41

Baig. 2015	peer	active	Inspecting the inside of your shoes	short	Community	Means, SD in each group	3.02	3.23	3.34	3.14	43	3.29	3.39	4.15	3.22	41
Baig. 2015	peer	active	Taking insulin injections as prescribed	short	Community	Means, SD in each group	6.08	2.06	6.86	0.53	43	5.89	2.26	7	0.001	41
Baig. 2015	peer	active	Taking oral medications as prescribed	short	Community	Means, SD in each group	6.06	2.25	6.77	1.19	43	6.69	1.33	6.86	0.85	41
Baig. 2015	peer	active	Testing your blood sugar	short	Community	Means, SD in each group	2.71	2.92	3.76	2.7	43	2.49	2.79	4.46	2.64	41
Rygg. 2012	expert	inactive	Patient activation (PAM)	short	Hospital	Means, SD in each group	71	14.4	72.8	14.9	73	72.4	15	71.7	16	73
Meng. 2016	expert	active	medication adherence	short	Hospital	Means, SD in each group	23.99	1.69	24.06	1.18	207	24.3	1.38	24.21	1.52	193
Meng. 2016	expert	active	response to symptom	short	Hospital	Means, SD in each group	10.04	1.65	10.54	1.6	207	9.95	2.02	10.56	1.62	193
Meng. 2016	expert	active	self monitoring	short	Hospital	Means, SD in each group	3.2	0.54	3.47	0.47	207	3.22	0.56	3.47	0.47	193
Meng. 2016	expert	active	symptom monitoring	short	Hospital	Means, SD in each group	8.69	2.47	9.5	2.47	207	8.41	2.49	8.88	2.51	193
Engelen. 2020	expert	inactive	PAM	long	Hospital	Means, SD in each group	60.6	14.6	61.9	15.3	78	56.2	13.9	56.4	15.7	96
Study name	provider	comparison	outcomes	time point	setting	data format	sample size(ex)	sample size(cont)	F or diff.							
Moriyama. 2009	expert	inactive	Exercise stage	long	Hospital	F for diff in change	42	23	0.231							
Hibbard. 2007	peer	inactive	regular exercise	short	Community	F for diff in change	244	235	9.2							
							diff. in means	sample size(ex)	sample size(cont)	independent groups p						
Lynch. 2014	expert	active	exercise	short	Community	Independent groups (difference, p)	0.6	29	26	0.17						
Lynch. 2014	expert	active	physical activity ,kcal/wk	short	Community	Independent groups (difference, p)	-603.4	29	26	0.01						
							mean(ex)	sample size(ex)	mean(cont.)	sample size(cont)	p					
Rujiwatthanakorn. 2011	expert	inactive	Aerobic exercise	short	Hospital	Independent groups (means, p)	58.23	50	37.92	46	0.001					
							mean difference(ex)	difference SD(ex)	sample size(ex)	mean difference(cont.)	difference SD(cont.)	sample size(cont)				
Lorig. 2009	peer	inactive	Aerobic exercise, min/wk ↑	short	Community	Mean change, SD difference in each group	9.7	115	161	-19.2	103	133				
Dongbo. 2003	peer	inactive	Aerobic exercise(minute/week)	short	Community	Mean change, SD difference in each group	27.93	175.51	406	2.68	136.51	319				
Dongbo. 2003	peer	inactive	Stretching and strengthening exercise(minutes/week)	short	Community	Mean change, SD difference in each group	3.54	64.1	411	4.44	55.13	321				
Liu. 2012	expert	inactive	Aerobic exercise (minutes/week)	long	Community	Mean change, SD difference in each group	23.11	176.71	98	-18.27	156.22	78				
Lorig. 2008	peer	inactive	Aerobic exercise(min/week) ↑	short	Community	Mean change, SD difference in each group	3.6	107	179	-3.47	115	173				
Lorig. 2008	peer	inactive	stretching/strength exercise(min/week) ↑	short	Community	Mean change, SD difference in each group	9.52	8.96	179	1.04	72.7	173				
Vinkers. 2014	expert	active	exercise	long	Community	Mean change, SD difference in each group	-0.09	1.41	45	-0.37	2.29	60				
Nishimura. 2017	expert	inactive	changes in exercise sunscale score	long	Hospital	Mean change, SD difference in each group	0.64	2.66	30	0.72	2.1	32				
							pre mean(ex)	pre SD(ex)	post mean(ex)	post SD(ex)	sample size(ex)	pre mean(cont.)	pre SD(cont.)	post mean(cont.)	post SD(cont.)	sample size(cont.)
Suwankruhasn. 2013	expert	inactive	Physical Activity	short	Hospital	Means, SD in each group	36.06	3.13	40.43	3.23	44	37.39	3.22	37.9	3.67	42
Trouilloud. 2013	expert	inactive	Physical Activity	short	Hospital	Means, SD in each group	1.71	1.71	2.99	2.04	99	2.28	2.17	2.7	1.99	99
Steed. 2005	expert	inactive	Exercise	short	Hospital	Means, SD in each group	2.25	2.21	3.78	2.04	50	2.8	2.2	3.02	2.4	50
Glasgow. 2012	expert	inactive	Physical activity(Cals/w;range 0-10000)	short	Hospital	Means, SD in each group	3989	3001.912	4410	3074.686	331	3915	3377.803	3704	3136.531	132
McGowan. 2011	peer	active	time doing physical exercise(0-4) ↑	short	Hospital	Means, SD in each group	0.5	1.18	0.72	0.41	82	0.75	0.61	0.74	0.58	152
McGowan. 2011	peer	active	time doing stretching/strengthening exercise(0-4) ↑	short	Hospital	Means, SD in each group	0.99	1.2	1.12	1.04	82	0.96	1.17	1.04	1.18	152
Baig. 2015	peer	active	Participating in 30 min or more physical activity	short	Community	Means, SD in each group	2.8	2.77	3.78	2.88	43	3.05	2.82	4	2.65	41
Baig. 2015	peer	active	Participating in a specific exercise session	short	Community	Means, SD in each group	1.34	2.15	2.9	2.91	43	1.63	2.34	1.61	2.23	41
Sadeghian. 2016	expert	active	Physical activity level	short	Hospital	Means, SD in each group	1.47	0.16	1.583	1.714	152	1.48	0.2	1.4893	1.812	154
Agarwal. 2019	peer	inactive	Physical activity	long	Hospital	Means, SD in each group	4.56	0.38	4.73	1.62	15	3.48	1.33	3.35	1.6	20
Engelen. 2020	expert	inactive	international physical activity Q_moderate	long	Hospital	Means, SD in each group	333	318	333	276	76	373	364	336	300	92
Engelen. 2020	expert	inactive	international physical activity Q_vogorous	long	Hospital	Means, SD in each group	145	197	155	210	76	114	170	161	214	96
Engelen. 2020	expert	inactive	international physical activity Q_walking	long	Hospital	Means, SD in each group	325	322	330	262	74	339	319	354	340	92
Meng. 2016	expert	active	physical activity	short	Hospital	Means, SD in each group	155.17	179.03	227.49	201.12	207	193.22	195.51	234.99	218.41	193
Study name	provider	comparison	outcomes	time point	setting	data format	sample size(ex)	sample size(cont)	F or diff.							
Hibbard. 2007	peer	inactive	manage stress in a healthy way	short	community	F for diff in change	244	235	1.3							
							mean(ex)	sample size(ex)	mean(cont.)	sample size(cont)	p					
Rujiwatthanakorn. 2011	expert	inactive	stress management	short	hospital	Independent groups (means, p)	49	50	47.96	46	0.297					
							chi square	total N								
Steed. 2005	expert	inactive	smoking(% given up)	short	hospital	Chi-squared for 2x2	1.71	21								
							ex events	ex total N	cont. events	cont. total N						
Sadeghian. 2016	expert	active	smoking	short	hospital	Cohort 2x2 (Events)	11	152	15	154						
							pre mean(ex)	pre SD(ex)	post mean(ex)	post SD(ex)	sample size(ex)	pre mean(cont.)	pre SD(cont.)	post mean(cont.)	post SD(cont.)	sample size(cont.)
Engelen. 2020	web base	inactive	nicotine dependence	short	hospital	Means, SD in each group	3.8	1.6	3.3	2.1	8	3.4	2.2	5	2.7	5
Study name	provider	comparison	outcomes	time point	setting	data format	sample size(ex)	sample size(cont)	F or diff.							
Steed. 2005	expert	inactive	smoking(% given up)	short	hospital	Chi-squared for 2x2	1.71	21								
							ex events	ex total N	cont. events	cont. total N						
Sadeghian. 2016	expert	active	smoking	short	hospital	Cohort 2x2 (Events)	11	152	15	154						

							pre mean(ex)	pre SD(ex)	post mean(ex)	post SD(ex)	sample size(ex)	pre mean(cont.)	pre SD(cont.)	post mean(cont.)	post SD(cont.)	sample size(cont.)
Engelen. 2020	web base	inactive	nicotine dependence	short	hospital	Means, SD in each group	3.8	1.6	3.3	2.1	8	3.4	2.2	5	2.7	5
